# Gut Microbiota in Irritable Bowel Syndrome and Inflammatory Bowel Disease: Differences in Pathophysiology, Biomarkers, and Treatment Implications

**DOI:** 10.3390/ph19050783

**Published:** 2026-05-17

**Authors:** Ploutarchos Pastras, Ioanna Aggeletopoulou, Vasiliki Psalti, Christos Triantos

**Affiliations:** Division of Gastroenterology, Department of Internal Medicine, University of Patras, 26504 Patras, Greece; ploutarchosp96@gmail.com (P.P.); iaggel@hotmail.com (I.A.); vassilikipsalti@gmail.com (V.P.)

**Keywords:** inflammatory bowel disease, inflammatory bowel syndrome, pathophysiology, biomarkers, treatment

## Abstract

Alterations in the intestinal microbiota have been implicated in both irritable bowel syndrome (IBS) and inflammatory bowel disease (IBD). However, their biological significance and therapeutic implications differ substantially between the two conditions. Although dysbiosis is a common feature, the mechanisms by which alterations in the microbiota contribute to disease pathophysiology and clinical expression are distinct. Some pathways are more prominent in IBS (e.g., the gut–brain axis), whereas others are more prominent in IBD (e.g., reduced microbial diversity). Equally important are pathways that appear to play a role exclusively in IBD [e.g., Adherent-invasive *Escherichia coli* (AIEC) and Paneth cells], as well as others that seem to be specific to IBS (e.g., mast cell activation). In IBD, microbiota changes are primarily linked to immune dysregulation, mucosal barrier impairment, and inflammation-driven pathways, whereas in IBS, they are mainly associated with functional disturbances mediated by neuroimmune signaling and microbial metabolites. Furthermore, several microbiome-associated biomarkers differ between these two diseases, and some are already assessed by international guidelines. Although the microbiota plays a key role in IBS and IBD pathophysiology, microbiome-based treatments remain limited, especially in IBD. There are clinically available treatments in IBS (e.g., rifaximin, low-FODMAP diet), but in IBD, only the probiotic VSL#3 is guideline-approved in ulcerative colitis pouchitis prophylaxis. Nevertheless, the dynamic nature of the microbiota continues to support the investigation of already studied (e.g., probiotics, fecal microbiota transplantation) and potential novel therapeutic approaches at the research level. The aim of this review is to compare the gut-microbiota-related pathophysiological pathways and biomarkers between IBS and IBD, to summarize the microbiome-related medications that have already been studied in both diseases, and to suggest new potential therapeutic options based on the gut microbiota.

## 1. Introduction

A wide variety of microorganisms inhabit the human gut and occupy distinct ecological niches along the gastrointestinal tract’s mucosal surface [1]. Collectively, these microorganisms constitute the gut microbiota, while the entire genetic material (genome) of these microorganisms, along with the environment in which they live (metabolites, molecules, and ecosystem conditions), is referred to as the gut microbiome [2]. The gut microbiome is the largest part of the human microbiome [3] and affects the neonatal immune system [4,5], immune regulation, homeostasis, and the modulation of the enteric nervous system (ENS) and the central nervous system (CNS) [6,7]. The relationship between the gut microbiota and the human host has been described as a bidirectional symbiosis [8]. Although this microbial community has been linked to several prevalent diseases [9], depending on the disease, different pathophysiological mechanisms have been identified as responsible for this phenomenon, e.g., disturbances of the microbiome-gut–brain axis in gastrointestinal diseases [10]. In this review, we will delve into the impact of the gut microbiota on two prevalent gastrointestinal disorders: Irritable Bowel Syndrome (IBS) and Inflammatory Bowel Disease (IBD).

IBS is a chronic disorder of the gut–brain interaction (DGBI) characterized by recurrent abdominal pain and bloating and associated with alterations in stool frequency or form [11]. The Rome Foundation recently conducted a multinational study across 33 countries, estimating IBS prevalence at approximately 3–5% worldwide [12]. IBS is classified into four types based on the predominant stool pattern: IBS-C (constipation–predominant), IBS-D (diarrhea-predominant), IBS-M (mixed type), and IBS-U (unclassified) [13]. The gut microbiota plays a central role in the dysfunction of the neuroimmune-gastrointestinal network involved in this disorder, partly by influencing neurotransmitter synthesis via microbial metabolites [14]. This is one of several pathophysiological mechanisms that contribute to IBS symptoms, leading to deterioration in patients’ quality of life [15].

Another gastrointestinal disease strongly influenced by the microbiome is IBD, which not only affects quality of life but also causes disability [16] and reduces life expectancy [17]. IBD is a complex group of diseases, mainly comprising Crohn’s disease (CD) and ulcerative colitis (UC). Its main symptoms include diarrhea, hematochezia, abdominal pain, fever, and malnutrition [18]. The burden of IBD is expected to remain high and continue to increase until 2050, due to its early onset and the recent rise in incidence [19]. Altered composition of the gut microbiota, known as dysbiosis, has been strongly associated with IBD etiology and pathogenesis [20].

Because the gut microbiota is involved in both IBS and IBD, and because both diseases place a substantial burden on patients, healthcare systems, and society, microbiome-based interventions, including probiotics and fecal microbiota transplantation (FMT), have been studied in both conditions, with variable results [21,22]. To date, comparative studies of the microbiome in these two diseases have focused on changes in the microbiota using a purely metagenomic, gene-targeted approach [23,24]. The novelty of the current review is its focus on when these microbiota changes cause the disease. The research gap it addresses is the translation of metagenomic findings into clinically relevant mechanisms, biomarkers, and therapeutic applications. This translation is achieved by categorizing each microbiota-associated pathophysiological mechanism, incorporating recently discovered mechanisms, and providing a unified framework that clarifies differences in the microbiome between the two diseases across clinically related pathophysiology, biomarkers, therapies, and future perspectives.

Thus, the aim of this review is to compare the gut-microbiota-related pathophysiological pathways and biomarkers in IBS and IBD, summarize microbiome-based therapeutic strategies that have been already studied in both diseases, and propose potential future therapeutic options based on the gut microbiota modulation.

## 2. Pathophysiology

### 2.1. Similar Pathways with Different Intensity

A common pathway through which the microbiome affects both IBS and IBD is dysbiosis [25,26]. Dysbiosis disrupts the intestinal epithelial barrier, alters microbial sensing by the innate immunity, and influences microbial metabolites through both its structural elements and functional consequences [25,26]. Several pathophysiological pathways are shared by the two diseases, although their intensity is different.

#### 2.1.1. Higher Intensity in IBD

Several dysbiotic and barrier-related features are consistently higher in IBD compared with IBS. Table 1 summarizes the higher intensity of these parameters in IBD compared with IBS.

Reduced microbial diversity compared with healthy individuals represents a well-established microbiota-related feature of IBD and a commonly reported, although less consistent, finding in IBS. An inflammatory gut environment increases oxygen and nitrogen-free radical levels, favoring the respiratory metabolism of aerobes (Enterobacteriaceae) and reducing anaerobic symbionts, including Firmicutes and Bacteroidetes. As a result, an inflammatory, dysbiotic environment is continually reinforced [27]. An increase in Proteobacteria and a reduction in *Faecalibacterium prausnitzii* have been observed in both diseases [27,28]. However, in IBD, the oxidative shift is consistently more pronounced due to chronic inflammation. A metagenomic analysis of 1792 participants showed that the microbiome composition had significant overlap between the two diseases but was distinct enough to distinguish IBD from IBS [23]. Microbiome variation in IBD is consistently greater than in healthy individuals, with CD showing more severe dysbiosis than UC [29]. On the other hand, some IBS patients do not exhibit detectable changes in fecal microbiota compared with healthy individuals [30]. In addition, one study found that *Faecalibacterium prausnitzii* levels were higher in healthy controls than in IBS patients, who showed a significant deficiency [31]. These findings may indicate that the reduction in microbial diversity is not present in all IBS cases.

In dysbiosis, the reduction in fiber-degrading bacteria, such as *Faecalibacterium prausnitzii*, leads to impaired production of short-chain fatty acids (SCFAs), especially butyrate. Although the exact mechanism is not yet fully elucidated, studies have shown that lower SCFA levels reduce receptor activation in gut epithelial cells, thereby impairing NLRP3 inflammasome activation and reducing interleukin 18 (IL-18) production. Thus, gut mucosal immunity is weakened [32]. At the same time, reduced SCFA availability and expansion of mucus-degrading bacteria may reduce the expression of tight junction proteins, including occludin, claudin-1 and ZO-1, thereby increasing paracellular permeability and disrupting the intestinal epithelial barrier, a recognized pathophysiological process [33,34]. Although this type of barrier dysfunction is found in both diseases, it appears to be more pronounced in IBD. Microbiome-SCFA relationships vary between IBS subtypes [35], whereas reduced SCFA production is a consistent finding in IBD [36]. In addition, IBD is characterized by complete barrier disruption with ulcerations, crypt destruction, and massive bacterial translocation, whereas IBS is associated with minor paracellular leakage without macroscopic mucosal damage [25].

Furthermore, SCFA levels increase tryptophan catabolism, reducing the availability of tryptophan for serotonin synthesis. Impaired serotonin production affects gut motility and visceral sensitivity [36]. However, early evidence suggests that the degree of tryptophan impairment differs between these two diseases. In IBD, serum tryptophan is more severely depleted, whereas in IBS, the depletion is less pronounced because metabolism tends to shift toward tryptamine production. In IBD, increased tryptophan catabolism decreases circulating tryptophan levels, and microbial conversion of tryptophan is associated with disease severity [29]. In IBS-D, increased levels of tryptophan and tryptamine have been observed, suggesting altered microbial tryptophan metabolism and potential involvement of serotonergic signaling in diarrhea-predominant symptoms [37].

Impaired intestinal epithelial barrier permeability is widely believed to increase the translocation of lipopolysaccharides (LPSs), peptidoglycan, and flagellin. These molecules are recognized by Toll-like receptors (TLRs) 4, 2, and 5, respectively, on macrophages and dendritic cells, leading to activation of nuclear factor kappa B (NF-κB). This triggers an inflammatory cascade, resulting in activation of the intestinal immune system [38]. In IBS, TLR4 and TLR5 expression are increased, promoting low-grade inflammatory cascades without macroscopic inflammation [25]. In contrast, IBD is characterized by marked NF-κB activation and a full Th1/Th17 immune response, whereas defective mucosal integrity triggers an immune response that sustains chronic inflammation [38].

Another pathophysiological pathway common to these two diseases, but more pronounced in IBD because of its greater inflammatory burden, is dysfunction of bile acid metabolism. Early evidence suggests that dysbiosis reduces the abundance of bacteria with 7α-dihydroxylation activity, leading to increased primary bile acids and decreased secondary bile acids in the gut lumen. This shift reduces anti-inflammatory signaling and contributes to visceral hypersensitivity [37,39].

#### 2.1.2. Higher Intensity in IBS

In both diseases, it is generally accepted that dysbiosis is associated with increased levels of *Ruminococcus gnavus* and *Ruminococcus torques*, which produce mucinases and degrade the mucus layer, thereby weakening the natural barrier between bacteria and epithelium. As a result, contact between bacteria and epithelium increases [25,27,38]. Interestingly, emerging data for a multicenter study with 1426 participants showed that *Ruminococcus Gnavus*-associated dysbiosis was more pronounced in IBS than in Ulcerative Colitis, with biofilm detected in 57% of IBS cases and 34% of UC cases [40].

Additionally, another well-established mechanism is that the microbiome affects the function of the gut–brain axis. It influences this axis through reduced vagal activation, which weakens the cholinergic anti-inflammatory pathway; altered microbial production of neurotransmitters, which affects brain processing of visceral pain; and increased cytokine levels, which activate the stress response and further dysbiosis [41]. These mechanisms contribute to both IBS and IBD. However, in IBS, the gut–brain axis is considered a primary pathophysiological mechanism [42]. In IBD, it is regarded as a secondary pathophysiological mechanism associated with chronic inflammation, contributing to functional symptoms during remission and to psychiatric comorbidity [43].

### 2.2. Different Microbiota-Associated Pathways Between the Two Diseases

**Figure 1:**
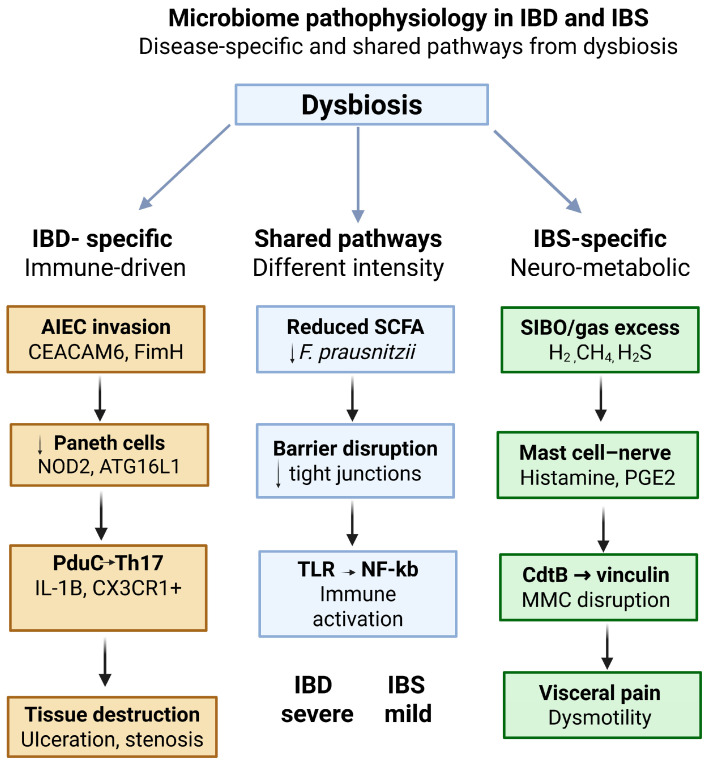
Microbiome Pathophysiology in Irritable Bowel Syndrome and Inflammatory Bowel Disease: Disease-Specific and Shared Pathways.

The gut microbiota influences IBS and IBD through multiple pathophysiological pathways in each disease. Some of these pathways have been observed only in IBD, whereas others have been observed only in IBS. Figure 1 shows the IBD-specific mechanisms, the IBS specific mechanisms and overlapping pathways.

The disease-specific mechanisms in IBD reflect a chronic, genetically determined interaction between the microbiome and the immune system [26]. In IBS, the specific mechanisms primarily involve neuromuscular and metabolic dysfunction, with the altered microbiome composition producing bioactive gases and mediators that directly affect mobility, sensation, and neuronal sensitization [25].

#### 2.2.1. Only in IBD

Adherent-invasive *Escherichia coli* (AIEC) strains are a specific finding in CD and have not been described in IBS. The generally accepted underlying mechanism is that the receptor CEACAM6 is overexpressed in the epithelium of CD patients [44]. AIEC binds to this receptor and invades gut epithelial cells. At the epithelial level, these bacteria can transcytose across the intestinal barrier into the lamina propria, where they may survive within macrophages [45]. Thus, they become resistant to autophagy, especially in carriers of ATG16L1/NOD2 mutations [46]. Their intracellular proliferation within the phagolysosome forms intracellular bacterial communities (IBCs), leading to chronic mucosal inflammation [47]. AIEC strains are frequently isolated from CD patients’ biopsies, and their ability to trigger intestinal inflammation supports their role as disease modifiers [46].

In addition, non-well-established data show that AIEC strains that metabolize large amounts of propanediol via the propanediol dehydratase (PduC) are enriched in the microbiome of CD patients and drive T-cell-mediated intestinal inflammation. CX3CR1+mononuclear phagocytes (MNPs) are essential for PduC-dependent induction of Th17 cells and IL-1β. PduC-mediated propionate production may act synergistically with LPS to promote chronic inflammatory colitis [48]. In animal models, PduC inhibition or fucose restriction reduces inflammation [48]. This mechanism appears to be specific to CD, as it depends on AIEC, which have not been described in IBS.

Another well-established cell type that is dysfunctional in CD is Paneth cells. In patients with NOD2 mutations, impaired NOD2 signaling reduces NF-κB activation in Paneth cells, contributing to defective antimicrobial peptide production and impaired microbial control [49]. Additionally, reduced autophagy is observed in patients with ATG16L1 mutations [46]. This genetic susceptibility impairs Paneth cell function and microbial sensing [49]. AIECs can exploit the deficit to evade autophagy, leading to bacterial overgrowth and chronic ileitis [46]. In IBS, Paneth cell dysfunction is not observed, and the associated genetic polymorphisms are not considered risk factors.

#### 2.2.2. Only in IBS

IBS-C is associated with increased methanogenesis and colonization by methanogenic archaea, recognized phenomenon referred to as intestinal methanogen overgrowth (IMO). Methane (CH4) has been directly associated with delayed intestinal transit in animal models [50]. IBS-C patients have been shown to have higher respiratory CH4 levels, which are associated with a greater abundance of fecal *Methanobrevibacter smithii* [50]. In contrast, in IBD, methanogenesis pathways were reduced compared with controls [23].

IBS-D patients had higher levels of respiratory hydrogen sulfide (H_2_S), associated with a greater abundance of H_2_S-producing bacteria, particularly *Fusobacterium* and *Desulfovibrio* [50]. Preclinical data suggest that H_2_S can trigger visceral pain reactions through TRPA1 receptor activation, thereby contributing to visceral hypersensitivity and abdominal pain in IBS. At the same time, H_2_S at high concentrations increases paracellular permeability in a dose-dependent manner [25]. In IBD, microbial H_2_S production pathways have been reported to be reduced [28].

This overproduction of gases can also be attributed to the generally recognized phenomenon of small intestinal bacterial overgrowth (SIBO). Impaired intestinal motility, particularly the disruption of the migrating myoelectric complex (MMC), causes intestinal stasis and bacterial overgrowth. This bacterial overgrowth may lead to excessive gas production, which can contribute to bloating, abdominal discomfort, and altered bowel habits, with IBS-C or IBS-D symptoms depending partly on the predominant gas produced [50]. SIBO is considered a manifestation of dysbiosis and is particularly common in IBS [51]. In IBD, SIBO may occur secondarily (e.g., after surgical resection or stenosis), but it is not considered a primary pathophysiological mechanism [52].

**Figure 2:**
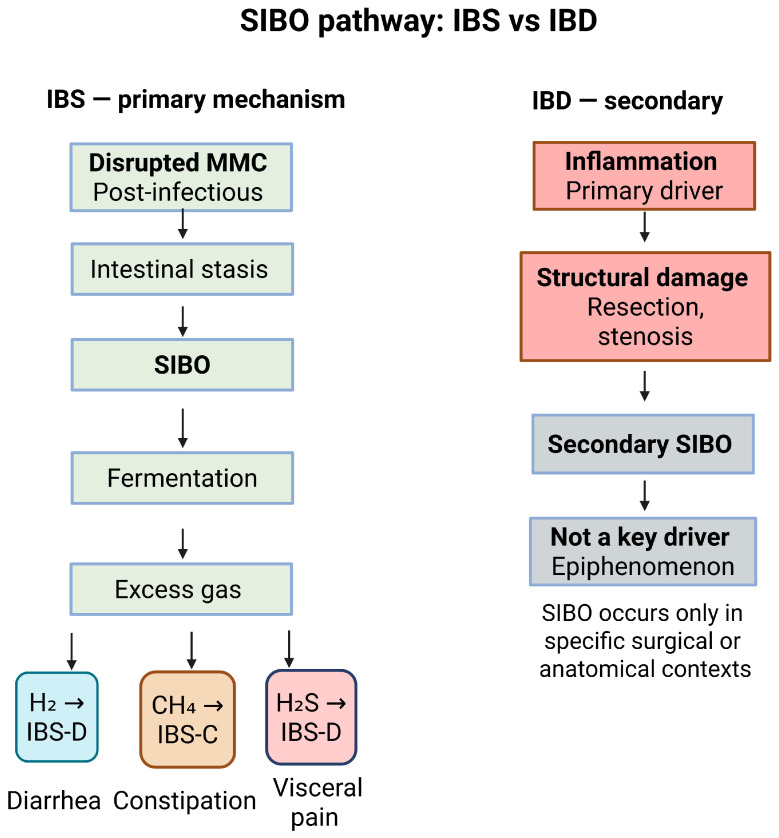
Differences in the SIBO-related pathophysiological pathway between IBS and IBD.

Furthermore, SIBO can be developed after a gastrointestinal infection and contribute to post-infection IBS (PI-IBS). Gram-negative bacteria, mainly *Campylobacter jejuni*, but also *Salmonella*, *Escherichia coli*, and *Shigella*, which cause acute infectious gastroenteritis, produce cytolethal distending toxin B (CdtB), which activates the host immune response and induces anti-CdtB antibodies [53]. Due to the molecular mimicry of CdtB and vinculin, a cell adhesion protein in the enteric nervous system, the host immune system also produces anti-vinculin autoantibodies. These autoantibodies damage the intermediate cells of Cajal (ICC) and enteric neurons, thereby disrupting the MMC [53]. This disruption causes intestinal stasis, SIBO, and PI-IBS [54]. In a study with 2375 participants, anti-CdtB and anti-vinculin levels were higher in IBS-D and lower in IBS-C, whereas levels in IBS-C did not differ significantly from those in healthy controls [54]. However, this mechanism has not been consistently reproduced across studies. An Australian study did not confirm that anti-CdtB and anti-vinculin antibodies could distinguish IBS-D from organic gastrointestinal disease [55]. In addition, a recent study found no significant differences in mean anti-CdtB and anti-vinculin levels between IBS subtypes and healthy controls [56]. Therefore, this mechanism is considered possible but remains controversial in IBS. In IBD, this autoimmune neuromuscular mechanism is not present. Figure 2 summarize the SIBO’s pathophysiological pathway in two diseases.

Another well-established pathophysiological pathway that appears to be specific to IBS is mast cell activation in close proximity to nerves, acting as a primary mechanism of visceral hypersensitivity. In IBS, increased translocation of microbial antigens, such as LPSs, through a disrupted intestinal barrier activates mast cells located near submucosal sensory nerve fibers. These mast cells release mediators [57], which sensitize visceral afferent nerves, and pain signals are transmitted via the spinal cord to the brain, thereby influencing the severity and frequency of abdominal pain [58]. At the same time, mast cells release nerve growth factor (NGF), which induce neuroplasticity and chronic visceral hypersensitivity [59]. In contrast, although mast cells are also activated in IBD, this occurs as part of a broader inflammatory response, and mast cell-driven visceral hypersensitivity is not considered the main pathogenic mechanism. In IBD, pain mainly results from inflammatory tissue damage, including edema, ulceration, and stenosis [60].

## 3. Biomarkers

**Figure 3:**
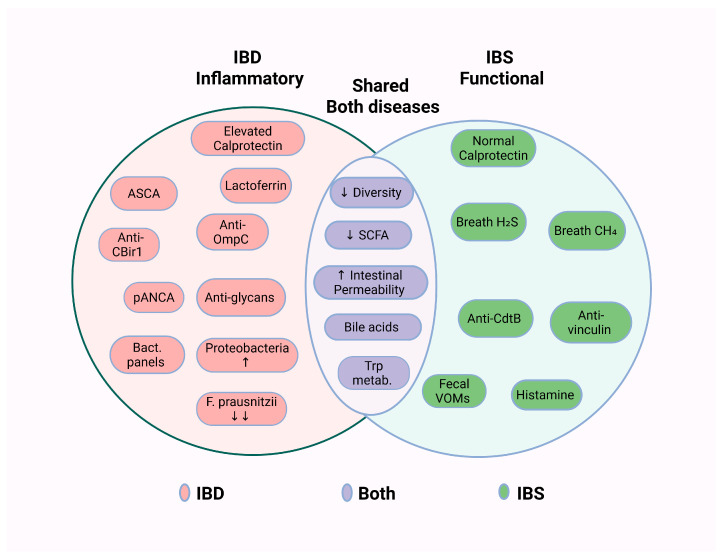
Microbiome-Associated Biomarkers and Pathophysiological Characteristics in Inflammatory Bowel Disease (IBD) vs. Irritable Bowel Syndrome (IBS).

Biomarkers are useful tools for diagnosing and monitoring many diseases, including IBS and IBD. Some biomarkers related to microbiome-associated pathophysiology are shared by both conditions, whereas others differ between IBS and IBD. Some are already used in clinical practice, while others are still at the research stage. A summary of the biomarkers discussed below is presented in Table 2 and Figure 3.

### 3.1. Common Biomarkers

Fecal calprotectin and fecal lactoferrin are not purely microbiome-related biomarkers, as they are neutrophil-derived proteins, but they reflect intestinal inflammation associated with dysbiosis. They have been reported to be among the most sensitive tests (0.97 and 0.94, respectively) for distinguishing IBD from non-IBD conditions, and they are also highly sensitive and specific for distinguishing IBD from IBS. These biomarkers are used in clinical practice and are elevated in IBD, whereas they are normal in the vast majority of patients with IBS [61]. The most recent guidelines of the European Crohn’s and Colitis Organization (ECCO) list fecal calprotectin as the most sensitive biomarker of intestinal inflammation in IBD and fecal lactoferrin as an alternative marker of intestinal inflammation in IBD, with a strong recommendation [62].

At the research level, SCFA and microbial diversity (α and β) have been investigated in both diseases. Reduced fecal SCFA levels have been reported in both IBD and IBS, although in IBS this may vary by subtype [58,63]. Similarly, reduced microbial diversity has been observed in both diseases, but it is generally more pronounced in IBD. Despite significant overlap, microbiome composition may still help distinguish IBD from IBS [28].

In addition, fecal bile acids may serve as biomarkers in both IBD and IBS. Patients with IBS and IBD have shown increased primary bile acids in fecal fluid, while secondary bile acids are significantly decreased [37,64]. In IBS and UC biofilms, bile acid accumulation has been correlated with fecal bile acid excretion, suggesting a diarrhea-related mechanism [40]. This biomarker appears altered in both diseases, but it remains a research biomarker and is not yet widely used in clinical practice.

Another biomarker currently under investigation is fecal tryptophan metabolites (indoles, kynurenine, tryptamine). In IBD, increased tryptophan catabolism depletes circulating tryptophan levels, while lower serum tryptophan and histidine levels have been consistently reported [65]. In IBS, tryptophan and tryptamine, bacterial metabolites involved in serotonergic signaling, have been found to be increased in the fecal samples of some patients, suggesting a potential role in altered gut–brain axis signaling and visceral sensitivity [37]. Overall, serum tryptophan appears reduced in IBD, whereas tryptamine appears increased in IBS.

Furthermore, bacterial translocation markers, such as LPS-binding protein, are elevated in IBD, whereas in IBS, increased levels have been reported only in certain subgroups and remain an inconsistent finding. Barrier disruption may lead to the release of bacterial metabolites and endotoxins into the circulation. This process can be promoted by bacterial infections, oxidative stress, a high-fat diet, alcohol exposure, and dysbiosis, and appears to be associated with the development of multiple diseases [66]. These markers are still considered research biomarkers and are not used in routine clinical practice.

### 3.2. IBD Biomarkers

Several biomarkers are clinically validated and used in routine practice for IBD, while comparable clinically established biomarkers are lacking for IBS. IgG and IgA antibodies against *Saccharomyces cerevisiae* mannan (ASCA) reflect loss of immune tolerance to microbial antigens. Elevated ASCA titers have been reported to identify CD patients with high specificity (96–100%) but low sensitivity (50%) [61]. Additionally, the antibodies against flagellin (a protein that is the main structural component of bacterial flagella) (anti-CBir1), and antibodies against the outer membrane porin C of *Escherichia coli* (anti-OmpC) have been associated with a more aggressive disease phenotype and more rapid progression to complications in CD [66]. Positivity for ASCA IgA, ASCA IgG, anti-OmpC, and anti-CBir1 has been detected in 65% of CD patients up to 6 years before diagnosis [67].

Anti-OmpC, along with ASCA and perinuclear antineutrophil cytoplasmic antibodies (pANCA), have been used to distinguish CD from UC, although their addition to ASCA and pANCA only marginally improves diagnostic accuracy [68]. pANCA are not directly microbial antibodies; their production is thought to be triggered by microbial antigens. Elevated pANCA levels are more common in UC and in CD with UC-like pancolitis. A meta-analysis of 60 studies estimated the sensitivity and specificity of the ASCA (+)/pANCA (−) pattern for CD at 55% and 93%, respectively [68].

In ASCA-negative patients, additional antibodies targeting glycans, that is, polysaccharides in the cell walls of microorganisms such as *Saccharomyces cerevisiae* and *Candida albicans*, may also be useful. These include anti-chitobioside carbohydrate antibodies (ACCAs), anti-laminaribioside carbohydrate antibodies (ALCAs), and anti-mannobioside carbohydrate antibodies (AMCAs). Each anti-glycan antibody has shown good specificity and a high positive predictive value for distinguishing CD from UC. In a small cohort of ASCA (−) patients, 44% were positive for ALCA or ACCA, yielding a sensitivity of 77% and a specificity of 90% [61].

However, ASCA, pANCA, anti-OmpC, anti-CBir1, and anti-glycan are included in the most recent ECCO guidelines, with a negative recommendation due to their limited precision and small added value to therapeutic decision-making [62].

As regards bacterial species panels, these remain research tools and have not yet been applied in clinical practice. A multi-species bacterial panel based on metagenomic data from 5979 fecal samples achieved an AUC of 0.91, with sensitivity of 79% and specificity of 92% for distinguishing IBD from IBS, showing numerically higher diagnostic performance than fecal calprotectin (AUC = 0.86) [69].

### 3.3. IBS Biomarkers

Breath gases are among the best-documented microbiome-related biomarkers in IBS and are used clinically to diagnose SIBO, intestinal methanogen overgrowth (IMO), and intestinal sulfidogen overgrowth (ISO), but they are not used in IBD. IBS-C patients have higher respiratory CH4 levels, associated with a greater abundance of *Methanobrevibacter smithii*, whereas IBS-D patients have higher H_2_S levels, associated with *Fusobacterium* and *Desulfovibrio* [50]. The most recent American College of Gastroenterology (ACG) guidelines for IBS management acknowledge the potential role of breath testing in selected clinical contexts, but emphasize that these tests should not be performed routinely in all patients [70].

At the research level, fecal volatile organic metabolites (VOMs) reflect microbial metabolic activity and can distinguish IBS from IBD using a panel of 11 core VOMs [71]. Fecal volatile organic compounds (VOCs) and metabolites, together with microbiome-related markers, have the potential to distinguish IBS and its subtypes from other gastrointestinal diseases [73].

Anti-CdtB and anti-vinculin are investigational biomarkers with inconsistent reproducibility. Anti-CdtB has shown a sensitivity of 43.7% and a specificity of 91.6% for distinguishing IBS from IBD [70]. Both anti-vinculin and anti-CdtB have been found to be significantly elevated in IBS patients compared to controls in some studies, but specificity was only 83.8% [72].

## 4. Treatment

Even if the microbiome plays a significant role in the pathophysiology of both diseases, the therapies used so far are broad-spectrum, not personalized, and pathophysiology pathway-specific. Several therapeutic interventions based on the microbiome have been studied and applied in patients with IBD and IBS. Some have shown benefits, some have produced controversial results, and some are not recommended for these diseases. A summary of the therapeutic implications of microbiome-targeted interventions according to current guidelines is presented in Table 3.

### 4.1. Probiotics

In IBS, probiotics are used in clinical practice as a potential treatment under certain conditions, whereas in IBD, they are generally not recommended, with a few exceptions. More specifically, in IBS, a meta-analysis of 53 randomized clinical trials showed that probiotic combinations had beneficial effects on global IBS symptoms and abdominal pain, although no definitive conclusions could be drawn regarding their overall efficacy [76]. The latest ACG clinical guidelines for IBS treatment provided a conditional recommendation, based on low-quality evidence, suggesting a possible benefit for bloating and gas, without recommending specific strains or species [70]. However, the latest American Gastroenterological Association (AGA)’s clinical guidelines on the role of probiotics in the management of gastrointestinal disorders concluded that there is insufficient evidence to support their use in CD, UC, or IBS. Therefore, the AGA suggested that patients taking probiotics for CD, UC, or IBS should consider discontinuing them [74].

An exception is the AGA’s conditional recommendation for an 8-strain probiotic combination of *Lactobacillus paracasei*, *Lactobacillus plantarum*, *Lactobacillus acidophilus*, *Lactobacillus delbrueckii*, *Bifidobacterium longum*, *Bifidobacterium breve*, *Bifidobacterium infantis*, and *Streptococcus salivarius* for preventing pouchitis in patients with UC after ileal pouch-anal anastomosis (IPPA) [74]. A double-blind, placebo-controlled trial showed that this highly concentrated probiotic preparation (VSL#3) significantly reduced the recurrence of chronic pouchitis and the onset of acute pouchitis after surgery [77]. VSL#3 has also been studied in mild-to-moderate UC. A double-blind, randomized, placebo-controlled study showed that a high dose of VSL#3, used as an adjunct to mesalamine, significantly increased remission rates compared with placebo [78]. However, current guidelines do not recommend VSL#3 for UC, and the available evidence remains limited to research studies. Another probiotic studied for UC remission is *Escherichia coli* Nissle 1917 (EcN). EcN at a dose of 200 mg per day was equivalent in efficacy to mesalazine 1500 mg per day in maintaining remission in UC [79]. The latest AGA guidelines found no clear benefit over mesalazine and did not recommend it, although this conclusion was based on non-inferiority studies [80]. Nevertheless, EcN is used clinically in several European countries, such as Germany, as an alternative to mesalazine, particularly because it lacks mesalamine’s adverse events [80]. Regarding Crohn’s disease, current data do not support the use of probiotics. In a randomized controlled trial, *Lactobacillus* GG was not effective in preventing relapse after surgical resection in patients with CD [81].

### 4.2. Antibiotics

Antibiotics, such as metronidazole, ciprofloxacin, and rifaximin, are used in CD for specific indications, such as peritoneal fistulas and pouchitis, but not as treatment for the disease itself through microbiome modification. Their use is not based on microbiome restoration but on their antimicrobial activity [82,83]. In contrast, antibiotics are used as a therapeutic option in IBS, particularly rifaximin. Rifaximin is a non-absorbable broad-spectrum antibiotic that targets the intestinal microbiota. Rifaximin causes modest changes in the microbiome, and its effectiveness may involve eubiotic actions, including regulation of inflammatory cytokines and intestinal permeability [84]. In two identically designed, phase 3 double-blind, placebo-controlled trials involving 1258 patients with IBS without constipation, rifaximin 550 mg three times per day for 2 weeks, followed by 10 weeks of follow-up, resulted in a significantly higher proportion of patients reporting adequate relief of overall IBS symptoms during the first 4 weeks after treatment compared with placebo (40.7% vs. 31.7% *p* < 0.001 in the two studies combined), as well as relief of bloating (40.4% vs. 30.3%, *p* < 0.001) [85]. In addition, a phase 3 retreatment study with IBS-D patients who had responded to an initial course of rifaximin but later relapsed, and randomized them to receive either rifaximin or a placebo, showed that a significantly higher proportion of patients in the rifaximin group responded to treatment compared with the placebo group (38.1% vs. 31.5%, *p* = 0.03) [86]. Additionally, a meta-analysis of 5 RCTs including 1803 patients found that rifaximin was associated with improvement in overall symptoms compared with placebo and demonstrated an excellent safety profile [76]. Thus, rifaximin receives a strong recommendation from the ACG for the treatment of global symptoms in IBS-D [70] and a conditional recommendation and moderate certainty from the AGA [87].

### 4.3. Low-FODMAP Diet

A diet low in fermentable oligosaccharides, disaccharides, monosaccharides, and polyols (FODMAPs) can modify the intestinal microbiome, with studies showing reductions in Bifidobacterium levels and total bacterial counts [27]. ACG recommends a trial of a low FODMAP diet to improve overall symptoms (strong recommendation, very low-quality evidence) [70]. This diet is also used clinically in IBD patients in remission with functional IBS-like symptoms. However, current IBD guidelines do not recommend it as a treatment for the underlying disease itself.

### 4.4. Fecal Microbiota Transplantation (FMT)

FMT transfers stool from a healthy donor into the patient’s colon, restoring the gut microbiota. It is the first-line treatment for recurrent *Clostridioides difficile* infection. In this infection, the restored microbiome interacts with the normal immune system (except in patients with comorbidities), whereas in IBD and IBS, this interaction remains abnormal after restoration [88]. Current guidelines do not recommend FMT for either disease. In IBS, data are insufficient, and clinical trial results have been conflicting. The AGA recommends against the use of FMT in IBS, except in the context of clinical trials [75]. In IBD, especially in UC, some RCTs have shown that FMT is significantly superior to placebo in inducing clinical and endoscopic remission in patients with mild to moderate disease [89,90]. However, despite these promising findings, the AGA also recommends against the use of FMT in UC and CD outside the context of clinical trials [75].

### 4.5. Prebiotics and Synbiotics

Prebiotics are dietary components that are not digested by endogenous enzymes in the human gastrointestinal tract and are fermented by the gut microbiota. They selectively stimulate the growth and activity of bacterial species, thereby positively affecting the host’s health [91]. The most common prebiotics include inulin, fructooligosaccharides (FOSs), galactooligosaccharides (GOSs), lactulose, and beta-glycans. These compounds are naturally found in onions, wheat, bananas, and other foods. Synbiotics are formulations that combine probiotics and prebiotics. Prebiotics support the viability and implantation of probiotics, acting synergistically [91]. Available data are limited for both diseases. At present, there are no recommendations for their use in either IBS or IBD, and studies remain at the research stage [74]. Table 4 shows the differences among probiotics, prebiotics, and synbiotics.

## 5. Discussion/Future Perspectives

### 5.1. General Discussion

The microbiome plays an important role in the pathophysiology of both IBD and IBS. Table 5 summarize the differences and similarities in gut microbiota pathophysiology between IBS and IBD.

Dysbiosis is the most common microbiota-associated pathophysiological mechanism in both diseases. Some related pathways appear to be more pronounced in IBD, including reduced microbial diversity, dysfunction of bile acid metabolism, decreased SCFA production, and immune activation through inflammatory signaling. Others seem more prominent in IBS, particularly dysregulation involving the gut–brain axis. In addition, some mechanisms appear to be disease-specific, as IBD is particularly associated with AIEC-related effects and Paneth cell dysfunction, whereas IBS is more closely linked to mast cell activation near nerves, methane production, and hydrogen sulfide production. There are differences in microbiota-associated biomarkers between the two diseases. In IBD, biomarkers with clinical use include ASCA, anti-OmpC, anti-CBir1, anti-glycan antibodies (ACCA, ALCA, AMCA), and pANCA. In IBS, breath gas CH4 and H_2_S have clinical utility. As regards treatment, only VSL#3 is recommended in pouchitis prophylaxis in UC, whereas rifaximin and a low-FODMAP diet are recommended for IBS.

### 5.2. Concluding Remarks

Even though the microbiome influences both IBD and IBS through several pathophysiological pathways, currently available microbiome-targeted therapeutic tools remain limited, particularly in IBD. This phenomenon may be explained by several factors. First, a direct causal relationship between dysbiosis and IBD has not been conclusively demonstrated in humans. Dysbiosis coexists with the disease, but it remains uncertain whether it is a cause or a consequence. Although gut bacteria often trigger immune activation, chronic inflammation itself can also reshape the microbiota and contribute to dysbiosis [88]. In addition, there is no specific dysbiotic pattern in IBD. The composition of the microbiota is strongly influenced by the presence or absence of mucosal inflammation, and dysbiosis is not specific to IBD, as it has also been associated with many unrelated diseases [92].

Another possible explanation is that antibiotics are often ineffective in IBD because the immune system, rather than the microbiome alone, may play the dominant role in disease progression. The direct causal relationship between pathogens and IBD-related inflammation remains under investigation, as some studies suggest that inflammation may also arise from broader disturbances in the microbial community [93]. This may explain why the most effective treatments for IBD currently target the immune system, such as infliximab, vedolizumab, ustekinumab, and tofacitinib, whereas none directly target the microbiome [94]. Furthermore, there is still no consensus on what defines a healthy microbiome. This reflects the complexity and highlights the influence of many factors, such as diet, geography, age, gender, lifestyle, and heredity [95]. Especially, DNA mutations and polymorphisms can modify both microbiota-related effects and disease action [49,96,97], making host–microbiome interactions and treatment responses highly individualized. This complexity is one of the reasons why no universally accepted definition of a healthy microbiome has yet been established [95]. Over the past decades, research and multi-omics techniques have advanced the field from mainly correlational observations toward a better understanding of causality in microbiome-based therapies. However, important gaps remain, including the lack of clinical guidelines, validated tools, and regulatory clarity [95].

### 5.3. Recommendations and Future Prospects

Despite these challenges, the microbiome remains a promising field for future therapeutic interventions in both IBD and IBS. The general trend is shifting from broad-spectrum approaches such as FMT and conventional probiotics to more precise and targeted therapies based on specific pathophysiological mechanisms.

Some emerging approaches may have potential applications for both diseases. Researchers have developed rationally designed microbial consortia, that is, combinations of specific bacterial strains intended to restore dysbiosis and induce anti-inflammatory effects, delivered as per os live biotherapeutics products (LBPs) [98]. The GUT-108 consortium was able to treat established colitis in gnotobiotic mice, reducing inflammatory markers to levels comparable to those of healthy controls while simultaneously increasing SCFA and secondary bile acids [99]. Secondary bile acids act as potent ligands for farnesoid X receptor (FXR) and G protein-coupled bile acid receptor 1 (TGR5), thereby regulating lipid metabolism and exerting immunoregulatory effects. TGR5 activation initiates an anti-inflammatory cascade via the cyclic adenosine monophosphate (cAMP)–protein kinase A (PKA) signaling pathway [99]. In mice that achieved remission, the baseline microbiota contained common microbes capable of regulating bile acid levels, and increased activation of FXR and TGR5 was associated with improved intestinal barrier function and reduced inflammation [100]. Rather than administering specific bile acids or synthetic agonists directly, an alternative therapeutic strategy may involve interventions at the microbial level, as the microbiome itself regulates bile acid homeostasis [100]. Another approach for both IBS and IBD is the therapeutic use of microbiome-derived metabolites. SCFAs, bile acid derivatives, and tryptophan metabolites, are increasingly recognized as bioactive molecules that regulate systemic processes and their therapeutic use, or the development of synthetic analogs, may represent a new therapeutic horizon [99]. Also, engineered probiotics developed through synthetic biology are being designed to recognize disease biomarkers, produce therapeutic molecules, and modulate immune responses with unprecedented specificity [99].

Other potential microbiome-based therapies appear to be more specific to IBD. AIECs represent a very promising target. Bacteriophages offer a way to selectively target specific bacteria involved in inflammation. A study showed that a per os cocktail of five *Klebsiella*-targeted lytic phages could selectively reduce *Klebsiella* populations in mice, thereby reducing inflammation without disrupting other microbes [98]. In contrast to antibiotics, bacteriophages exhibit high specificity, often even at the strain level, thereby minimizing unwanted off-target effects on the microbiome [101]. In addition, molecules that inhibit fimbrial adhesin H (FimH) binding to CEACAM6 may prevent AIEC colonization without affecting the rest of the microbiome, although these are still at the research stage [46]. Other investigational approaches include PduC inhibitors, that reduce inflammation in animal models [48], therapies of Paneth cell restoration, which may reverse dysbiosis in ileal CD [49], and in situ microbiome gene editing. Recent data suggest that targeted base editing of bacteria in the gut of mice may become possible, opening the way for precise modification of pathogenic traits without eliminating entire species [102].

As regards IBS, potential microbiome-based therapeutic targets include anti-methanogen therapies through selective inhibition of *M. smithii* [50], interventions that reduce H_2_S production in order to alleviate visceral pain [25], immunomodulatory approaches targeting anti-vinculin pathways to reduce SIBO [53], and newer H1 antihistamines that are currently under investigation [57].

In conclusion, future research should focus on identifying disease-specific microbial signatures, validating microbiome-based biomarkers for clinical use, and conducting rigorous trials of microbiota-targeted therapies before any routine clinical application in IBS or IBD.

## Figures and Tables

**Table 1:** Pathophysiological Features More Pronounced in Inflammatory Bowel Disease (IBD) than in Irritable Bowel Syndrome (IBS).

Feature	IBD	IBS
Oxidative shift/ increase in protobacteria	More pronounced due to chronic inflammation; stable increase in Proteobacteria/Enterobacteriaceae	Can be present in some patients, but is less consistent
Reduction in *Faecalibacterium prausnitzii*	Consistent reduction	Reduced in some patients, but not in all cases
Reduced microbial diversity	Consistently greater reduction; Dysbiosis is more severe, especially in CD than UC	Some patients show no clear fecal microbiota changes compared with healthy controls
Reduced SCFA production (especially butyrate)	Consistent reduction	Microbiome–SCFA relationships vary by subtype
Intestinal barrier disruption	Much more severe: ulceration, crypt destruction, and marked bacterial translocation	Usually, mild paracellular leakage without macroscopic mucosal damage

Abbreviations: IBD: Inflammatory Bowel Disease; IBS: Irritable Bowel Syndrome; CD: Crohn’s Disease; UC: Ulcerative Colitis; SCFA: Short-Chain Fatty Acid.

**Table 2:** Microbiome-Associated Biomarkers in Inflammatory Bowel Disease (IBD) vs. IrritableBowel Syndrome (IBS).

Biomarker	IBD	IBS	Clinical Use (According to Current Guidelines)	References
Fecal Calprotectin	Increased	Normal	Yes	[61,62]
Fecal Lactoferrin	Increased	Normal	Yes	[61,62]
SCFA	Decreased	Decreased	-	[58,63]
Microbial Diversity	Decreased	Decreased	-	[58,63]
Primary Fecal Bile Acids	Increased	Increased	-	[37,40,64]
Tryptophan Metabolites	Decreased	Increased	-	[37,65]
Bacterial Translocation Markers	Increased	Increased	-	[66]
ASCA	Increased (CD)	-	No	[61]
Anti-OmpC	Increased (CD)	-	No	[66,67]
Anti-CBir1	Increased (CD)	-	No	[66,67]
Anti-Glycans (ACCA, ALCA, AMCA)	Increased (CD)	-	No	[61]
pANCA	Increased (UC)	-	No	[68]
Bacterial Species Panels	Increased *	- *	-	[69]
Breath Gas CH_4_	-	Increased (IBS-C)	Yes (no as a routine)	[50]
Breath Gas H_2_S	-	Increased (IBS-D)	Yes (no as a routine)	[70]
Fecal VOMs	-	Yes	-	[71]
Anti-Cdtb	-	Yes	-	[70,72]
Anti-Vinculin	-	Yes	-	[72]

* Much more pronounced in IBD than in IBS (dysbiosis). Abbreviations: IBD: Inflammatory Bowel Disease; IBS: Irritable Bowel Syndrome; SCFA: Short-Chain Fatty Acid; ASCA: Anti-Saccharomyces cerevisiae Antibodies; CD: Crohn’s Disease; anti-OmpC: Anti-Outer Membrane Protein C; Anti-CBir1: Anti-Flagellin; ACCA: Anti-Chitobioside Carbohydrate Antibody; ALCA: Anti-Laminaribioside Carbohydrate Antibody; AMCA: Anti-Mannobioside Carbohydrate Antibody; pANCA: Perinuclear Anti-Neutrophil Cytoplasmic Antibody, UC: Ulcerative Colitis; CH_4_: Methane; IBS-C: Irritable Bowel Syndrome–Constipation; H_2_S: Hydrogen Sulfide; IBS-D: Irritable Bowel Syndrome–Diarrhea; VOMs: Volatile Organic Metabolites; anti-CdtB: Anti-Cytolethal Distending Toxin B.

**Table 3:** Microbiome-Associated Treatments in Inflammatory Bowel Disease (IBD) vs. Irritable Bowel Syndrome (IBS) according to clinical guidelines.

Therapy	IBD	IBS	References
Probiotics	VSL#3 only in UC pouchitis prophylaxis	Potential treatment only for bloating and gas	[70,74]
Antibiotics	NA	Rifaximin in IBS-D	[70]
Low FODMAP	NA	Improving overall symptoms	[70]
FMT	NA	NA	[75]
Prebiotic/Symbiotics	NA	NA	[74]

Abbreviations: IBD: Inflammatory Bowel Disease; IBS: Irritable Bowel Syndrome; VSL#3: an 8-strain probiotic combination of *Lactobacillus paracasei*, *Lactobacillus plantarum*, *Lactobacillus acidophilus*, *Lactobacillus delbrueckii*, *Bifidobacterium longum*, *Bifidobacterium breve*, *Bifidobacterium infantis*, and *Streptococcus salivarius*; UC: Ulcerative Colitis; IBS-D: Irritable Bowel Syndrome–Diarrhea; FMT: Fecal Microbiota Transplantation; FODMAP: Fermentable Oligosaccharides, Disaccharides, Monosaccharides, and Polyols; NA: not applicable.

**Table 4:** Differences between probiotics, Prebiotics, and Synbiotics in Inflammatory Bowel Disease (IBD) and Irritable Bowel Syndrome (IBS).

	Probiotics	Prebiotics	Synbiotics
Definition	Bacteria or Yeasts	Indigestible fiber/oligosaccharides	Probiotics + Prebiotics
Microbiome Effect	Temporary but Immediate addition of beneficial bacteria to the microbiome, inhibiting competitive pathogens	Prolonged feeding The selective existence of beneficial bacteria increases SCFA production	Synergistic action: prolonged probiotic action
IBD	VSL#3 in the prevention of pouchitis in UC patients with IPPA EcN as an adjunct to mesalamine in UC remission	limited data	limited data
IBS	Potential benefit in bloating and gas	limited data	limited data
Guidelines	IBD: Only VSL#3 [74] (AGA 2020) IBS: conditional recommendation [70] (ACG 2021)	-	-

Abbreviations: IBD: Inflammatory Bowel Disease; IBS: Irritable Bowel Syndrome; SCFA: Short-Chain Fatty Acid; UC: Ulcerative Colitis; VSL#3: an 8-strain probiotic combination of *Lactobacillus paracasei*, *Lactobacillus plantarum*, *Lactobacillus acidophilus*, *Lactobacillus delbrueckii*, *Bifidobacterium longum*, *Bifidobacterium breve*, *Bifidobacterium infantis*, and *Streptococcus salivarius*; IPPA: ileal pouch-anal anastomosis; EcN: *Escherichia coli* Nissle 1917.

**Table 5:** Summary of Key Differences and Similarities in Gut Microbiota Pathophysiology between Inflammatory Bowel Disease (IBD) and Irritable Bowel Syndrome (IBS).

	IBD	IBS
Dysbiosis Severity	Severe and Consistent (more pronounced in CD than UC)	Mild to Moderate
Microbial Diversity	Consistently and Significantly Reduced	Not universally reduced across all patients or subtypes
*Faecalibacterium prausnitzii* levels	Severely Depleted (especially in CD)	Moderately Reduced
SCFA production	Consistently Impaired	Variable reduction depending on subtype (more in IBS-D)
Barrier Disruption	Complete with macroscopic mucosal damage	Minor paracellular leakage without macroscopic mucosal damage
SIBO	Secondarily	Primary Pathophysiological mechanism
Inflammation	Marked NF-kB activation with full Th1/Th17 immune response. Massive neutrophil and T-cell recruitment. Tissue destruction including ulceration, edema, stenosis and fistula	Low-grade mucosal immune activation. Increased TLR4/TLR5 expression without macroscopic inflammation. Mast cell activation near nerves drives visceral hypersensitivity rather than tissue destruction
Fecal Calprotectin	Significantly Elevated	Normal (vast majority of patients)
FMT	Promising in UC but not recommended in guidelines	Insufficient and conflicting data

Abbreviations: IBD: Inflammatory Bowel Disease; IBS: Irritable Bowel Syndrome; UC: Ulcerative Colitis; CD: Crohn’s Disease, SCFA: Short-Chain Fatty Acid; IBS-D: Irritable Bowel Syndrome—Diarrhea; SIBO: Small Intestinal Bacterial Overgrowth; FMT: Fecal Microbiota Transplantation.

## Data Availability

No new data were created or analyzed in this study. Data sharing is not applicable to this article.
